# Do we understand the intervention? What complex intervention research can teach us for the evaluation of clinical ethics support services (CESS)

**DOI:** 10.1186/s12910-019-0381-y

**Published:** 2019-07-15

**Authors:** Jan Schildmann, Stephan Nadolny, Joschka Haltaufderheide, Marjolein Gysels, Jochen Vollmann, Claudia Bausewein

**Affiliations:** 1 Institute for History and Ethics of Medicine, Martin Luther University Halle-Wittenberg, Magdeburger Str. 8, 06112 Halle, Germany; 2 Institute for educational and health-care research in the health sector (InBVG), Bielefeld University of Applied Sciences, Interaktion 1, 33619 Bielefeld, Germany; 3 University of Applied Sciences for Diakonia, Bethelweg 8, 33617 Bielefeld, Germany; 4 Institute for Medical Ethics and History of Medicine, Ruhr-University Bochum, Markstr. 258a, 44779 Bochum, Germany; 5 Centre for Social Science and Global Health, University of Amsterdam, AHTC, Tower C4, Paasheuvelweg 25, 1105 BP Amsterdam, Netherlands; 6 Department of Palliative Medicine, Munich University Hospital, LMU Munich, Marchioninistr. 15, 81377 Munich, Germany

**Keywords:** Clinical ethics support services, Complex intervention research, Conceptual framework, Ethics consultation, Evaluation research

## Abstract

**Background:**

Evaluating clinical ethics support services (CESS) has been hailed as important research task. At the same time, there is considerable debate about how to evaluate CESS appropriately. The criticism, which has been aired, refers to normative as well as empirical aspects of evaluating CESS.

**Main body:**

In this paper, we argue that a first necessary step for progress is to better understand the intervention(s) in CESS. Tools of complex intervention research methodology may provide relevant means in this respect. In a first step, we introduce principles of “complex intervention research” and show how CESS fulfil the criteria of “complex interventions”. In a second step, we develop a generic “conceptual framework” for “ethics consultation on request” as standard for many forms of ethics consultation in clinical ethics practice. We apply this conceptual framework to the model of “bioethics mediation” to make explicit the specific structural and procedural elements of this form of ethics consultation on request. In a final step we conduct a comparative analysis of two different types of CESS, which have been subject to evaluation research: “proactive ethics consultation” and “moral case deliberation” and discuss implications for evaluating both types of CESS.

**Conclusion:**

To make explicit different premises of implemented CESS interventions by means of conceptual frameworks can inform the search for sound empirical evaluation of CESS. In addition, such work provides a starting point for further reflection about what it means to offer “good” CESS.

## Background

Ethics consultation and other forms of clinical ethics support services (CESS) have been developed to support healthcare professionals, patients and relatives by analyzing value conflicts related to clinical practice [1]. The term “clinical ethics support services” is used with regards to different kinds of institutionalized services within healthcare organizations, which support healthcare professionals and institutions in dealing with moral issues. In this paper, we limit the term to prospective interventions which support ethical decision-making in the case of moral conflicts related to treatment of an individual patient. CESS has been supported by professional [2–4] and regulatory bodies [50, 51] and has been increasingly implemented in various countries [5–8]. In addition, emphasis has been placed on the quality of CESS more recently [9]. However, there has been a paucity of evidence on the outcomes of CESS [10, 11], and considerable controversy regarding the contribution of CESS to clinical practice [12, 13] and the role of normative theory [14]. Given the complex normative and empirical challenges researchers face when evaluating outcomes of CESS [12, 15–18], it is surprising that there is only a little conceptual methodological work on this topic [11, 17, 19]. There has been specifically little input, so far, by experts in health research methods with knowledge and skills relevant to sound evaluation of interventions in healthcare [20, 21]. Collaboration between ethicists and health service researchers seems to be needed to fill in current gaps regarding a normative and empirically informed sound evaluation of CESS outcomes [18]. More outcomes research of CESS, in turn, is necessary for practical and theoretical reasons. Firstly, it is important to be able to describe the impact of CESS on clinical practice. This is true for positive and possible negative effects. Secondly, findings from outcomes research serve as an important rationale for economic decision-making in healthcare. As long as clinical ethicists request funding within the healthcare system, they need to be prepared to demonstrate the outcomes of their work. Thirdly, findings of outcomes research can trigger important normative questions concerning the justification of priorities (e.g. regarding underlying goals) of a particular CESS [10, 16, 18, 19, 22–25].

The need for more methodological work on CESS evaluation and our understanding that such an investigation needs input particularly from health research were the starting points for a larger interdisciplinary study on the empirical and normative challenges associated with evaluating CESS which has received funding by the German Federal Ministry for Education and Research (Grant No. 01KG1404). The research encompasses an ongoing Cochrane review on the effectiveness of CESS as well as conceptual and methodological analysis [26]. In this paper, we summarize the findings of the latter part of the project and focus on a study in which we explored the possible contribution of “complex intervention research” to inform outcomes research in CESS. In a first step, we will introduce the concept of “complex interventions” and show how CESS fulfils the criteria for such a type of intervention. In a next step, we present the development of a “conceptual framework,” a graphic illustration of active ingredients of complex interventions of CESS, in the form of ethics consultation on request. We subsequently apply this model to two published models of CESS: “proactive ethics consultation” and “moral case deliberation” and compare both models regarding their mode of action. In the final section, we discuss in which ways the analysis of CESS by means of a conceptual framework can inform outcomes evaluation research.

## Main text

### CESS as complex interventions

From a health service research perspective, an important task is to clarify how an intervention used in healthcare might work. When discussing this topic within the interdisciplinary working group, we realized that while there is great confidence that CESS does work, there is little elaboration on exactly how CESS might work. A published comparison of different CESS models in Europe [27] suggests that the outcome of CESS hinges on several factors. In this sense, the mode of action of CESS can be described as “complex”. Against this background, we tested whether CESS may qualify as what is called a “complex intervention” in health service research. The Medical Research Council (MRC) describes these types of interventions as follows: “Complex interventions are built up from a number of components, which may act both independently and interdependently. The greater the difficulty in defining precisely what exactly are the ‘active ingredients’ of an intervention and how they relate to each other, the greater the likelihood that you are dealing with a complex intervention” [28]. An example of a “complex” intervention is an outpatient palliative care service; different professionals provide support to patients near the end of life on different levels and by different means. On the other end of the spectrum are “simple” interventions, such as the injection of insulin. In this case, clinicians and patients can easily standardize the procedure and it is well-known what is happening regarding effects for which mechanisms.

The MRC names several indicators which can help to identify whether an intervention is complex or not in the revised version of the MRC framework on complex interventions [29]. Below, five dimensions of complexity according to the MRC framework are listed [29].

1. Number of and interactions between components within the experimental and control interventions.
2. Number and difficulty of behaviors required by those delivering or receiving the intervention.
3. Number of groups or organizational levels targeted by the intervention.
4. Degree of flexibility or tailoring of the intervention permitted.
5. Number and variability of outcomes.

Applying these criteria indicates that CESS is clearly a complex intervention for the following reasons: 1) It involves interactions and communication between different – and heterogeneous – stakeholders on a personal, professional and organizational level. 2) Different groups involved in CESS may refer to different professional codes of ethics. This requires skills by those who offer CESS, as well as those who request and participate in CESS. In addition, specific professional knowledge and competences need to be considered when searching for solutions to ethical conflicts in clinical practice. 3) CESS targets not only different groups of professionals, but also patients and relatives in the hospital. Consequently, there may be tensions between the groups, for example, regarding the goals of CESS. 4) There is a considerable variation of structural and procedural elements of CESS, as this has been shown, among others, by the multidimensional evaluation of different European CESS models [27]. 5) There is a considerable number and different types of outcomes which have been used for evaluating CESS [10, 17].

In order to be able to evaluate complex interventions, it is crucial to understand how the intervention might work [29]. It is particularly necessary to understand its assumptions, active components which influence the outcome, and related interactions between different elements of the intervention [28–30]. One tool that can assist with understanding complex interventions regarding their active components is a so-called “conceptual framework” [31]. In the next step, we will introduce this tool and describe its development for a generic CESS model.

### Development of a conceptual framework for ethics consultation on request

A “conceptual framework” is a graphic description of a complex system and/or the related structures and processes. The goal of using conceptual frameworks within the context of complex intervention research is to identify elements and relationships within that system which are deemed to be important for the functioning and outcomes of the system [32]. Given our overall interest in outcomes evaluation research in CESS, conceived as “complex intervention”, we developed a conceptual framework, which captures typical elements of CESS. While a number of different types of CESS have been described, we used request-based types of prospective ethical case consultations as starting point for our work. According to descriptions in the literature (e.g. [33–36]) as well as on our own practical experience in clinical ethics, this type of CESS has been implemented in many healthcare institutions as a “standard approach” to CESS [5–8] . The aim of development of the conceptual framework was to better understand which “generic” elements of (request-based) CESS might be relevant for making a decision about appropriate outcomes. We used a template for a so-called “process type” of conceptual framework published by Rohwer et al. [37] for the purpose of development. Developing this conceptual framework for CESS followed a six-stage process adapted from the literature [31, 38–40]: 1) A *scoping review* searching for existing conceptual frameworks in CESS. This scoping review did not generate any relevant findings. 2) The members of the interdisciplinary team performed a comprehensive, written, *structured brainstorming exercise*. The task was to collect different possible outcomes, settings, theoretical assumptions, target groups, etc. relevant to CESS. As part of this structured brainstorming exercise, the information was sorted around overarching topics related to outcomes research of CESS. This was followed by a round where team members voiced ideas on the topics identified. The collected topics and related comments were discussed within the group and transformed into a mind map. This step proved to be particularly important to clarify the different disciplinary and personal perspectives on CESS within the interdisciplinary team. 3) We *prioritized* those aspects of CESS deemed particularly relevant for a better understanding of CESS and its outcomes. 4) Through *reviewing the literature* on CESS evaluation, we validated and expanded the results of the preceding steps. 5. In the *(Re-)modelling phase,* we developed a first draft of the conceptual framework informed by the template mentioned earlier [37]. The focus of this step was on explaining structural and procedural elements of a generic CESS model that was deemed particularly relevant for the evaluation of its outcomes. As part of a *peer review,* the draft model was presented and discussed in a research colloquium with researchers from various disciplines at the Institute for Medical Ethics and History of Medicine in Bochum and external experts (see acknowledgements). After some remodelling in line with the feedback, the conceptual framework was presented at different workshops with researchers involved in the evaluation of moral case deliberation (MCD) and ethics consultation.

1. Searching for existing conceptual frameworks
2. Structured brainstorming
3. Prioritization
4. (Theoretical) Validation
5. (Re-)Modelling
6. Peer review

**Fig. 1:**
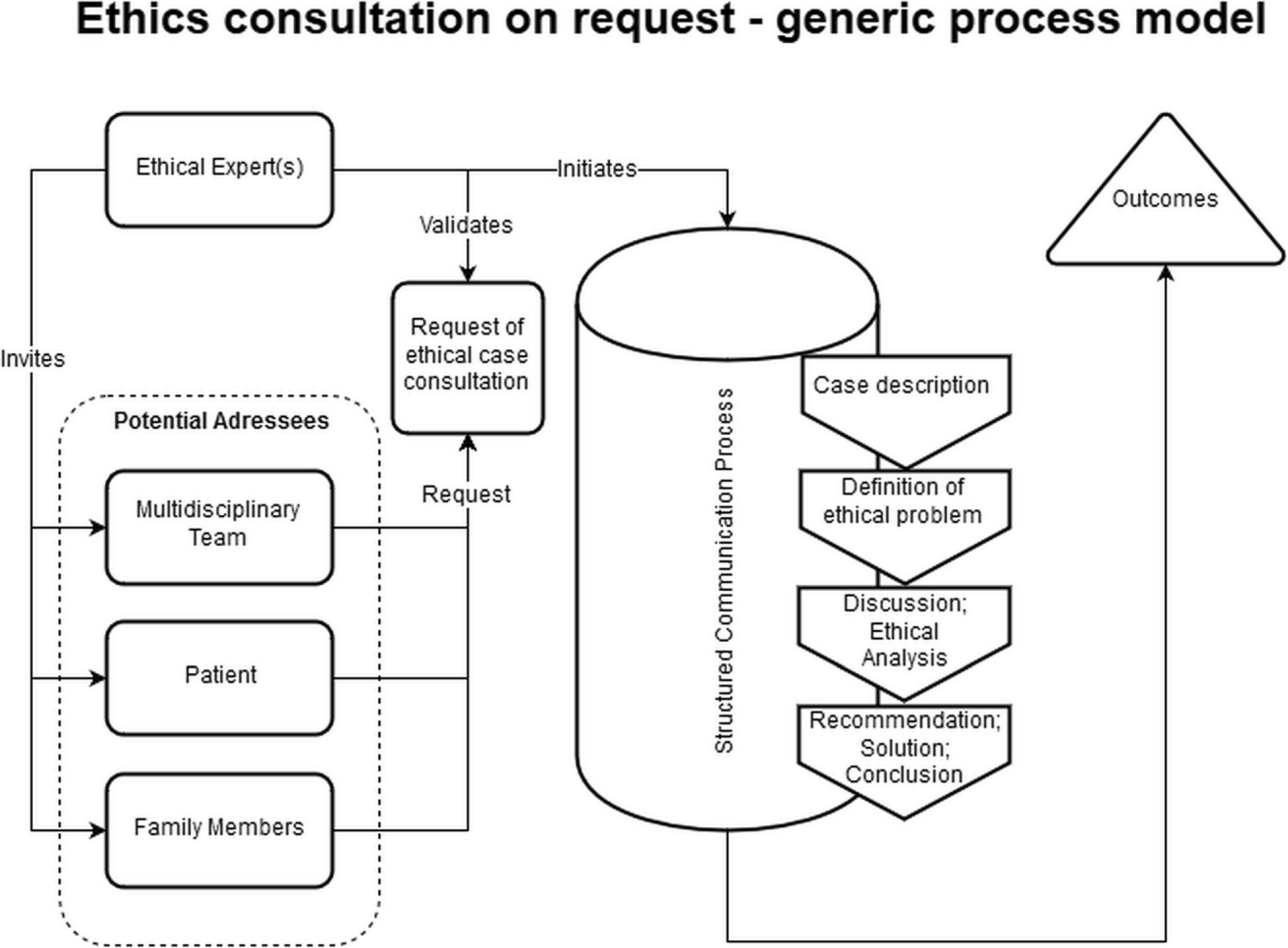
Conceptual framework (process model) of ethics consultation on request

**Fig. 2:**
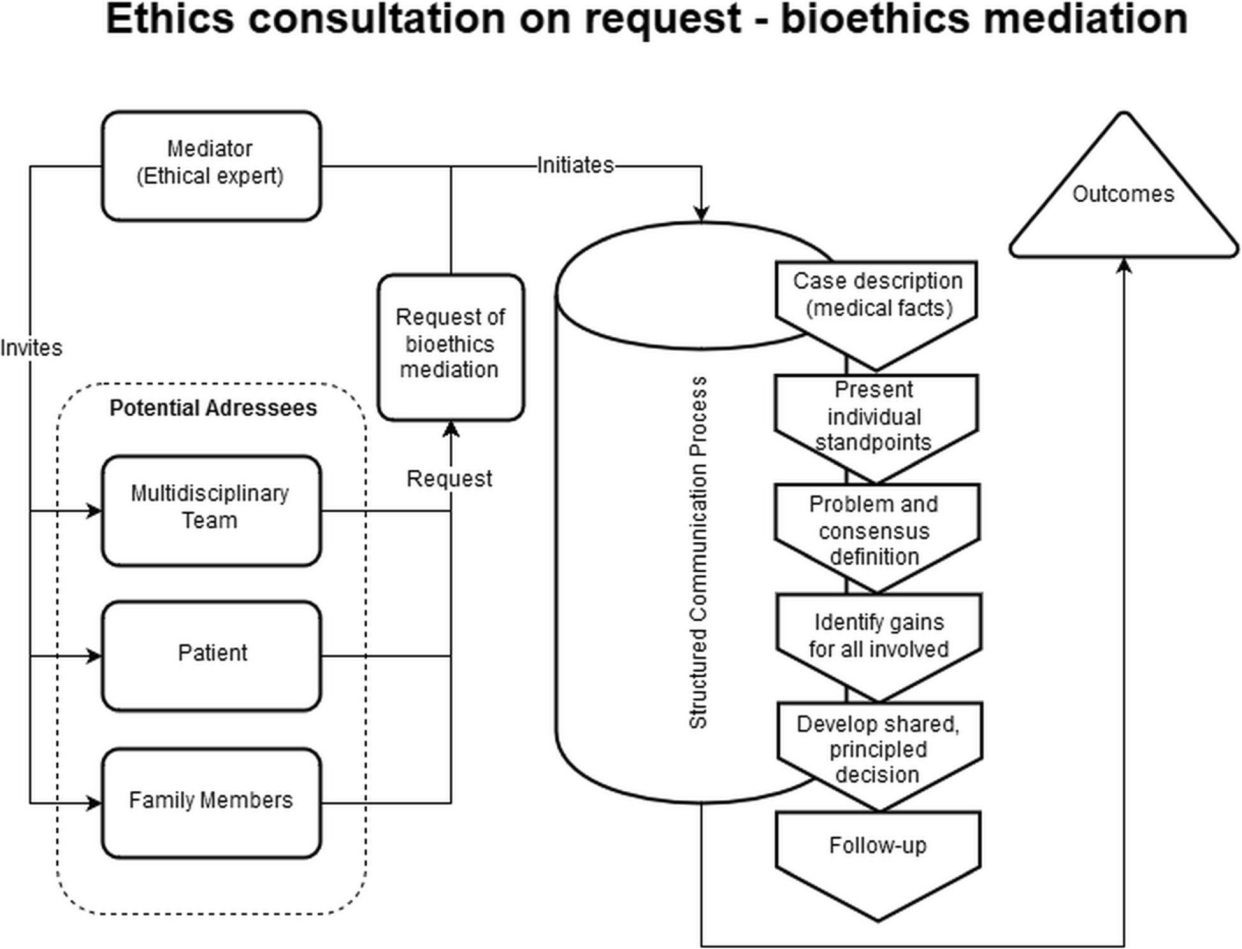
Conceptual framework (process model) of ethics consultation on request in the form of bioethics mediation according to the description by Schlairet (2009) [41]

Figure 1 presents the resulting conceptual framework in the form of a generic process model of ethics consultation on request. While there are variations between request-based approaches in CESS practice (see 27) this model according to our analysis captures many elements of “standard” CESS activities in the field. Figure 2 shows a conceptual framework for one specific form of request-based ethics consultation, namely bioethics mediation. As indicated in both figures, CESS is initiated after a request (by one or several groups) in combination with a decision by the clinical ethicist(s) whether the request qualifies for conducting a CESS. After inviting one or more groups to take part, a structured communication process takes place. The starting point is a description of the case, and at the end there is a conclusion and/or recommendation for further action related to the moral issue analyzed [20]. The specific elements of the structured communication process for bioethics mediation (see Fig. 2) have been reconstructed on the basis of the publication by Schlairet [41]. In line with the published account of this request-based model of ethics consultation our conceptual framework contains a “follow up” process element which takes place subsequent to the consultation process (see Fig. 2).

**Fig. 3:**
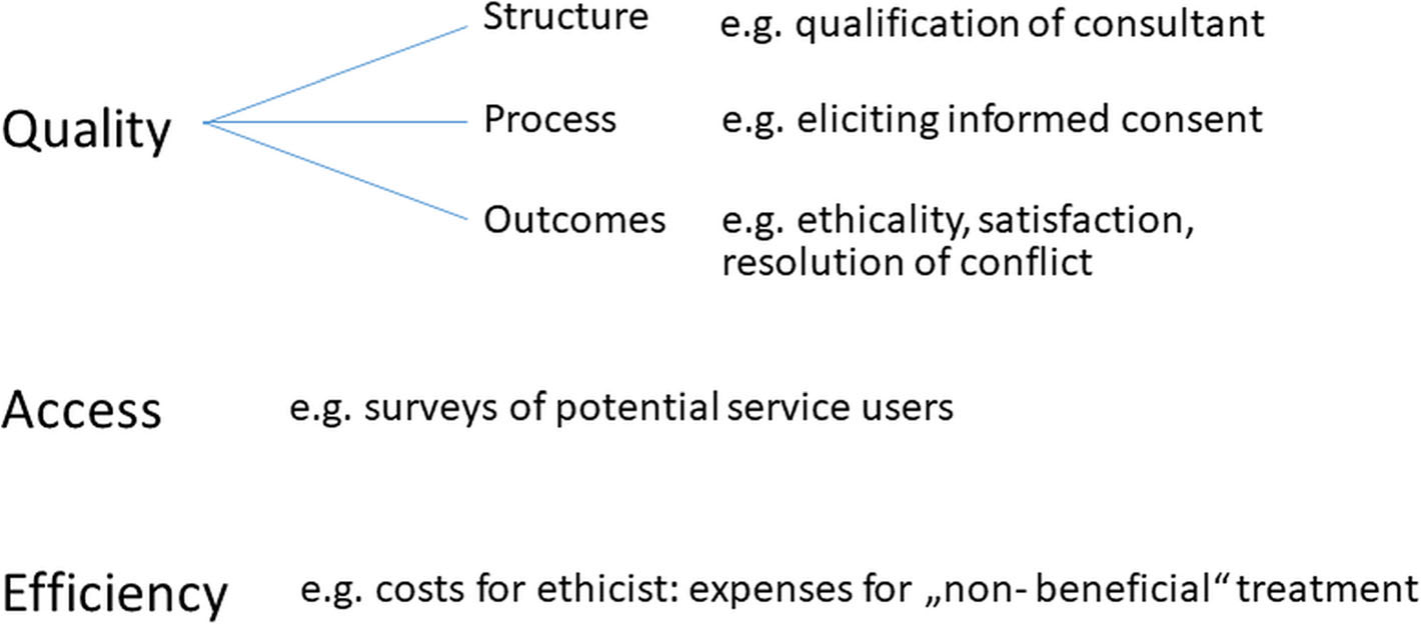
Structure, process and outcome criteria for CESS adapted from Fox (1996) [19]

With regards to potential outcomes of request-based ethics consultation, a list of outcomes used in controlled trials has been developed as part of a systematic review [26]. The outcomes in Table 1 show a broad range of endpoints reflecting different aspects of quality of care such as perceptions of different user groups (e.g. satisfaction) or impact on actual clinical practice (e.g. non-beneficial treatment). In addition, further evaluation criteria including domains of quality other than outcomes (i.e. structure or process) are listed in Fig. 3.

**Table 1:** Selection of outcomes of CESS as investigated in evaluation studies

Domain of outcome	Endpoint(s)
Duration of treatment	Days of receiving nutrition/hydration/ventilation
	Length of stay
	Time to/until complete of ethics intervention
Education	Education of professionals/family/patients
	Satisfaction with education
	Usefulness for learning from a difficult cast
Hospital costs	Hospital costs
Impact on clinical practice	Agreement with reached decision
	Usefulness to create agreement/improve cooperation/share responsibility
	Consultation resulting in consequences
	Changes in treatment plan
	Consensus reached
	Likelihood to request again/recommend ethics intervention
Mortality	Mortality
Quality of ethics consultant	Explain legal issues
	Identify key issues and options in care
	Support participants
	Perceived role of the ethicist
Satisfaction and helpfulness (consultation)	Clearness of the advice
	Informativeness
	Supportiveness
	Stressfulness
	Fairness
	Helpfulness in analyzing/identifying/resolving/clarifying ethical issue
	Helpfulness with improving communication/mediating disputes/providing emotional support
	Respectfulness of the patients/healthcare providers values
	Usefulness of being better equipped to deal with such cases/clarifying values at risk
	Satisfaction as perceived by patient/family member/surrogates/healthcare providers
Satisfaction and helpfulness (treatment)	Satisfaction as perceived by patient/family member/surrogates/healthcare providers
	Helpfulness with medical treatment
	Overall effectiveness of the ethics service’s involvement in the case
	Usefulness for getting support/in reaching better ethical decision/for broader discussion/getting advice/getting external perspective

### Moral case deliberation and proactive ethics intervention

A comparative analysis of potentially active factors of different types of CESS by means of conceptual frameworks

In recent years different study groups have reported data on evaluation research of specific CESS. Based on a systematic review on studies, which report data on the effectiveness of CESS [26] we have selected two types of CESS as candidates for further analysis of potentially active factors which are relevant from the perspective of evaluation research – moral case deliberation [42] and proactive ethics consultation [43]. We selected these types because both are well described in the respective evaluation research papers and in related publications [44, 45]. Moreover, an initial assessment of both models suggested that they are sufficiently distinct, and that the distinguishing elements and processes, which are relevant from an evaluation perspective, can be shown by means of analysis with conceptual frameworks. In the following, we describe both CESS models in accordance with the findings of our analysis using conceptual frameworks. Subsequently, we analyze how far the differences detected may be relevant regarding evaluating outcomes of the respective CESS models.

#### Moral case deliberation

**Fig. 4:**
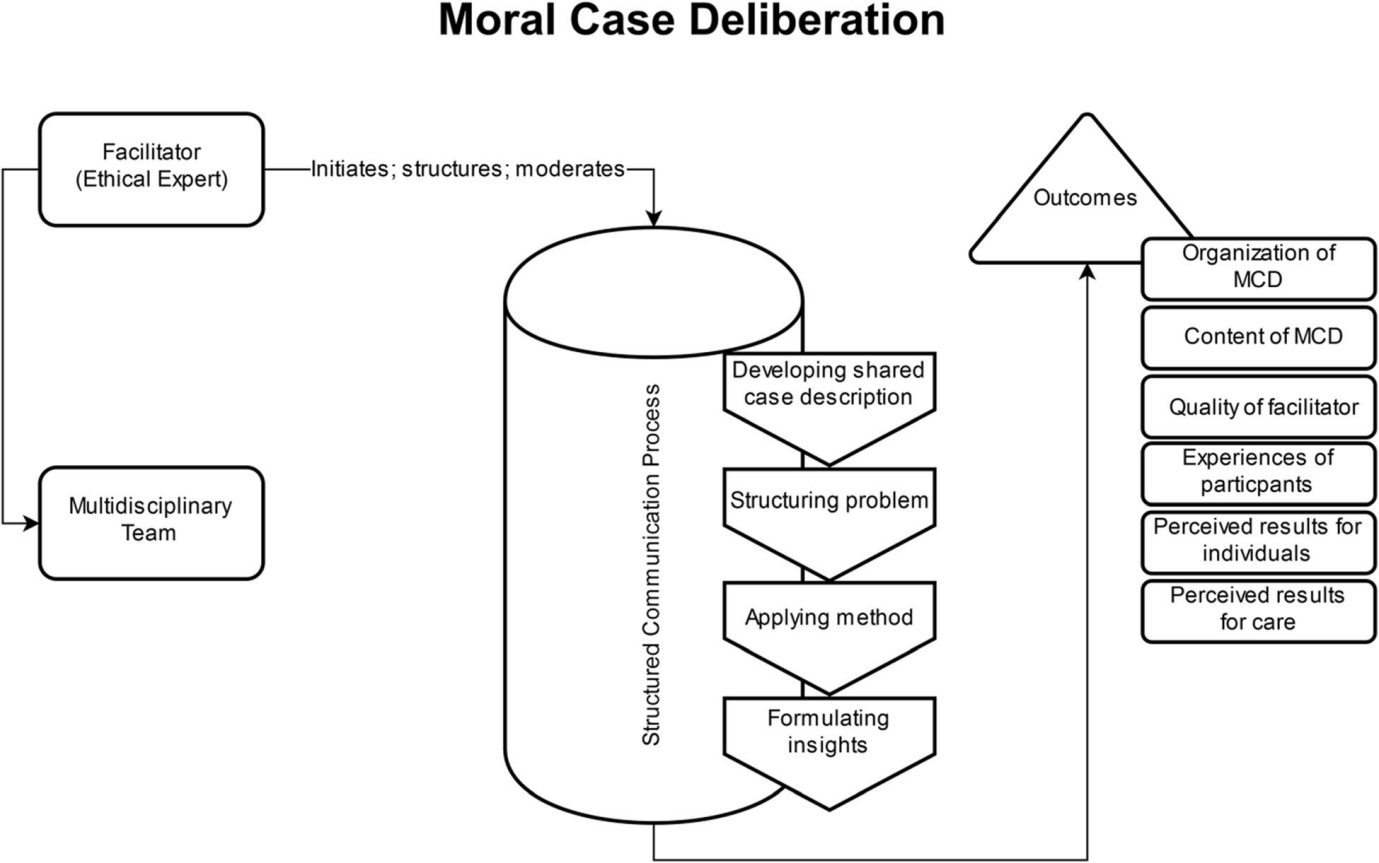
Conceptual framework (process model) of moral case deliberation, according to the description by Jansens et al. (2015) [42]

Janssens et al. (2015) described that MCD can be understood as a structured and methodological deliberation on how MCD participants perceive morally good (organization of) care. Moral case deliberation is designed to support healthcare professionals when being confronted with moral dilemmas and questions [42]. There is usually a series of MCD sessions scheduled in agreement with the management of a health institution. As shown in Fig. 4, a facilitator invites participants for the individual MCD session. The facilitator is an employee of the health institution, in this case an organization with 20 care centers for people with a range of conditions, who has received training in MCD. Although most participants have a nursing background, other health professionals and stakeholders may also participate. Respect for each other’s moral views and a willingness to critically assess one’s own moral convictions is required by MCD participants [42]. Both, the process and content of the critical reflection within MCD, aim to increase the caregivers’ awareness of the moral aspects of their daily work [42]. Finally, “on the basis of the judgement the group gradually steps back to the abstract level of reflection in order to formulate a philosophical insight” [46]. The structured communication process developed as part of the generic model of ethics consultation (see Fig. 1) may be adapted by different “conversation methods”, which, in the case of the paper cited, were “the dilemma method and the Socratic dialogue” [42]. The participants submit the cases discussed prior to the session and the respective “case topics” have to relate to a real-life experience of the case presenter with a genuine concern or uncertainty regarding what was morally right to do. In MCD, a described characteristic of the facilitator is their non-directive manner. Accordingly, the quality of the deliberation process and the meaningfulness of the moral issues are in the focus of the structured communication process [44]. Figure 4 shows the finding of the analysis of MCD by means of a conceptual framework.

#### Proactive ethics consultation

**Fig. 5:**
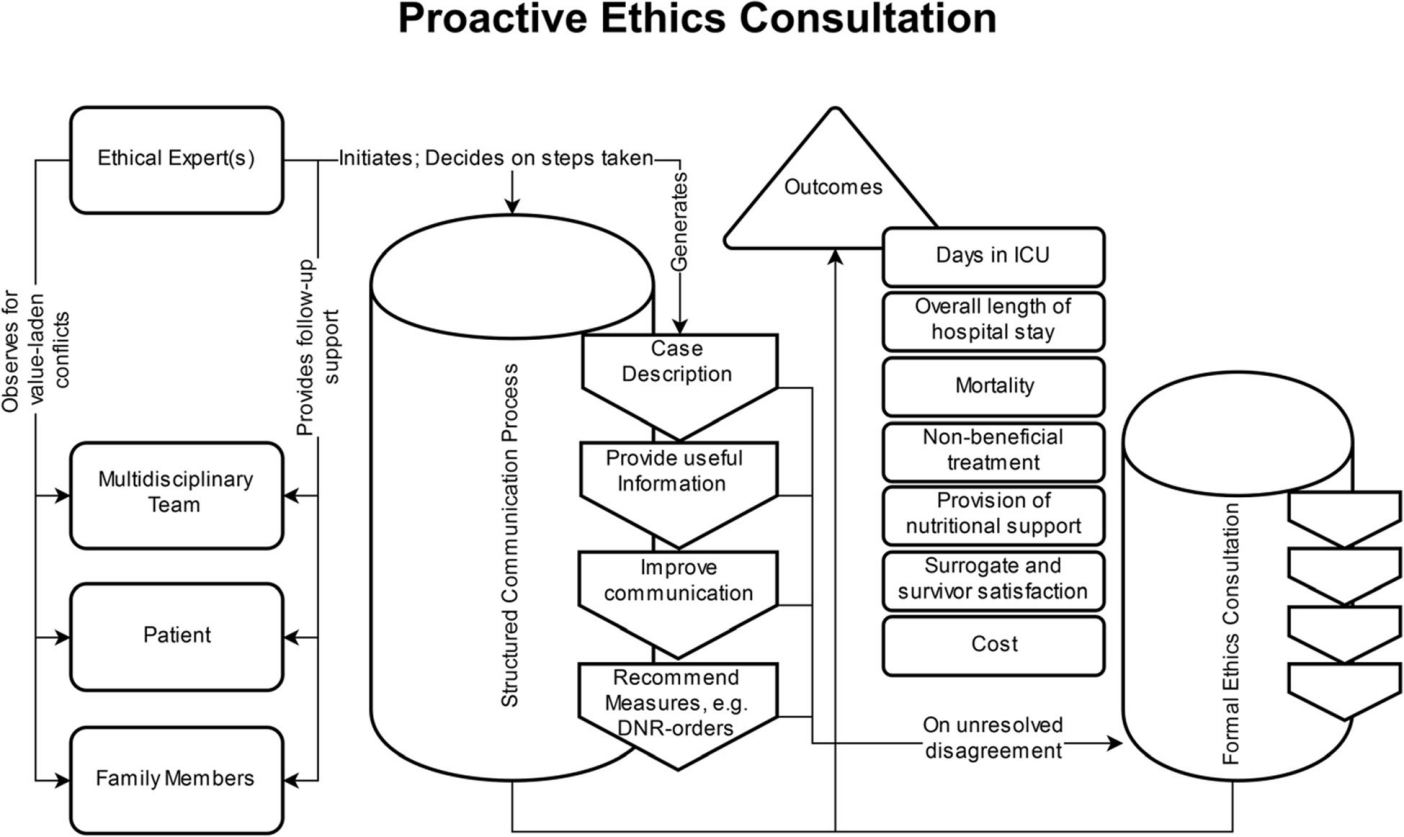
Process model which reconstructs ethics consultation in its proactive and escalated form, as described by Andereck et al. (2014) [43]

As shown in Fig. 5, a proactive ethics consultation described by Andereck et al. [43] differs in numerous ways from MCD. In this model of CESS, the ethical expert is a professional actively involved in daily clinical care who is able to determine “the patient’s medical condition, their decision-making capacity and preferences relative to treatment” [43]. The training of the clinical ethicist encompasses a Master’s and a PhD in bioethics, a clinical fellowship and three years of experience in ethics consultation [43]. One trigger for the ethics consultant to become active is a length of stay of five days in the intensive care unit. This is based on the authors’ experience that longer stays in the intensive care unit are associated with ethical challenges. In such a situation, the ethics consultant reviews the clinical case and contacts the relevant parties (i.e. patients, relatives, health professionals) to explore whether there are any value-related issues and specific factors known to contribute to ethical conflicts (e.g. lack of decisional capacity, religious constraints). When a potential ethical problem is recognized, the clinical ethicist uses different strategies to prevent or solve the ethical conflicts. Examples are the provision of relevant information, attempts to improve communication between parties, dealing with emotional discomfort or raising discussions about do-not-resuscitate orders [43]. If these or comparable interventions on the side of the clinical ethicist are not perceived to be sufficient, a “formal ethics consultation” is suggested. In any event, the ethical expert will follow up the case to discharge and document the respective activities in the medical record.

#### Differences in CESS and their relevance for outcomes research

The comparative analysis of MCD and proactive ethics consultation by means of conceptual frameworks indicates that there are some differences on the structure and process level. While it is known that CESS models work differently, the above analysis by means of conceptual frameworks provides in-depth insights concerning where CESS differs precisely within the system and its related processes. Regarding the two CESS models analyzed, these differences can be linked to the *targets and goals* of each CESS, which underlie the different setups. In addition, the CESS models ascribe differing *roles to the ethicists*, which seem to reflect different *understandings of expertise*.

Moral case deliberation is targeted primarily at health professionals, whereas proactive ethics consultation is targeted at patients, relatives and health professionals involved in a particular clinical case. The setups are in line with the different goals of each CESS model. While proactive ethics consultation seeks to prevent ethical conflicts and aims at addressing upcoming conflicts at an early stage, MCD focuses on reaching a shared understanding and finding possible solutions to moral conflicts associated with the care of one or several patients among participating health professionals. In accordance with the different targets and goals, the ascribed role of the ethicist also differs considerably in each CESS type. In proactive ethics consultation, the clinical ethicist works closely with or even within ongoing clinical practice. This role presupposes that the clinical ethicist has an objective set of knowledge and skills, which enables her or him to identify and solve moral conflicts. Such a role and understanding of expertise of the clinical ethicist contrasts with the description of the ethics facilitator in MCD. In the latter, the main task of the ethicist seems to be to create space for moral deliberation with some distance to daily practice. In such an environment, it is the task (and the expertise) of the ethicist to reconstruct moral perceptions and views of practitioners. Notably, there are also differences with respect to processes, which appear similar at first sight. One example refers to “description of the case”. While for both types of CESS this is an important step, different parties might interpret it differently. In MCD as described in Janssens et al. (2015), participants with background in healthcare present the case. However, as known from empirical research, a case and underlying reasons for actions related to a case may depend on who describes it [47]. In this respect, case description as starting point for the ethical analysis may differ considerably in different CESS models.

The admittedly brief comparative analysis of both CESS models already points to consequences for evaluation research. Before spelling these out, it should be noted that in evaluation research on CESS (and other interventions) two different types of evaluation can be distinguished. On the one hand there is summative evaluation, which is often carried out by external experts to assess whether pre-defined aims have been reached [48, 49]. Accordingly summative evaluation requires clearly definable and justifiable outcomes. On the other hand formative evaluation – often done as kind of self-evaluation (e.g. by consultants) follows the idea of evaluation as integrated feedback mechanism that can be used to control, alter or steer an interventions’ process on basis of evaluative experiences during the process [48].

The potential benefit of reconstructing CESS models by means of conceptual frameworks may provide additional value for both types of evaluation. With regards to summative evaluation, the admittedly brief comparative analysis of both CESS models already points to consequences regarding selection and use of “appropriate” outcomes. While it is beyond the scope of this article to provide an in-depth analysis of what means “appropriate” in this context taking into account normative as well as empirical criteria, one concept which may inform the respective research is the different notion of expertise which has been built into both types of CESS and which can be demonstrated graphically by means of the conceptual frameworks. Concerning MCD, expertise with regards to moral issues in practice is vested with those working in the practice. Accordingly, a choice of outcome criteria which inform about the impact of CESS on the perceptions of health professionals and their understanding and handling of moral conflicts would be consistent with the structure and process detected in this CESS model. In fact, the EURO-MCD evaluation tool developed by Svantesson et al. (2014) [21] to evaluate MCD focuses on domains such as “enhanced collaboration”, “enhanced emotional support” and “improved moral reflexivity”. In contrast in proactive ethics consultation expertise with regards to the detection and solution of ethical conflicts seems to be located in the ethics consultant. In addition, this expertise seems to be linked to some kind of improvement of patient care. Accordingly, the outcomes suitable for proactive ethics consultation by means of the conceptual framework suggests an approach of evaluation much more closely linked to the “ethical expertise” (which would need to be defined in more detail) and the impact of the work of the consultant on the care for individual practice. The active and partly preventive approach could be linked to outcomes such as decline of legal complaints or reduction of decisional conflicts on the side of patients and/or relatives. Given the closer link to clinical practice, this may also mean that chosen health-related outcomes differ depending on the clinical context (e.g. intensive care versus psychiatry) in which such a CESS model is implemented.

In addition to its contribution to summative evaluation, conceptual frameworks may provide value also by being a part of formative evaluation processes. Ethics consultants may for example want to use conceptual frameworks of their consultation model to reconstruct how it actually works and whether the running process is (still) in line with their predefined aims and goals. In a more detailed version, such frameworks could not only be used to reconstruct the way a CESS model works but also the way CESS worked with regards to an individual case. In this sense, Conceptual frameworks could be part of formative feedback elements to help consultants to keep all structural elements in sight, to identify unintended consequences of - or influences on the process and to optimize results of the process. A detailed retrospective analysis of how a CESS worked with regards to a case could also inform the future work of a CESS in the next case and improve quality. A brief overview of questions and checks in evaluation research where the use of conceptual frameworks may be beneficial is given in Table 2. Furthermore, and on a more general level, such an approach may inform educational programs on CESS and other interventions in the field of clinical ethics (e.g. teaching). This is because the change of perspective towards a (complex) intervention and respective elements can stimulate our thinking about the “best” way to set up and perform respective interventions. Furthermore, reconstructing CESS and other clinical ethics interventions by means of conceptual frameworks can stimulate normative analysis. This is because the analysis can make the often cryptonormative elements built within structures and elements of CESS explicit. By bringing up these issues, conceptual frameworks can also serve as a starting point for a reflection about “good” or “right” within the context of CESS and other ethical interventions directed towards medical practice.

**Table 2:** Potential benefits of conceptual frameworks for questions in evaluation research

	Question	Benefit
Summative evaluation	• What are possible outcomes of certain interventions?	• Use frameworks to develop in-depth understanding of complex interventions like CESS
		• Use frameworks to identify unknown or unclear elements and how they impact
	• Is this outcome appropriate, given the structure of this intervention?	• Use frameworks to define and justify appropriate outcomes for evaluation in line with aims and goals.
	• What kinds of outcomes can be used to evaluate different interventions?	• Use frameworks to develop generic outcomes suitable for all kinds of CESS
Formative evaluation	• Do elements of the intervention work as expected?	• Use frameworks as feedback and steering mechanism during process to alter and optimize intervention
	• Are there any unintended side-effects/consequences as result of the intervention?	
	• Are there any unclear influences, effects on elements of CESS?	
	• Were performed actions in line with aims and goals?	• Use frameworks to retrospectively analyse a single case
Additional use	Inform teaching	

## Limitations

Limitations of this analysis of CESS models by means of conceptual frameworks are, first, that the type of conceptual framework focuses on structural and procedural aspects of CESS but does not consider more systemic and contextual aspects possibly relevant for evaluating outcomes. Second, the conceptual framework in its presented form does not make the theoretical and professional backgrounds of those providing ethics consultation or facilitating MCD explicit. Third, the analyses in this paper are limited to CESS models focusing on ethical issues related to a single or a series of clinical cases. It is not known whether such an analysis would also be helpful for other models of CESS or ethics support services within other contexts. Fourth, this work only demonstrates that we can distinguish types of CESS meaningful in rather different groups, such as MCD and pro-active CESS. Next to the prima facie differences of both approaches, both types had been selected because of existing evaluation studies on outcomes with regard to these types of CESS. Given that there are several other broadly used CESS models, such as CESS focusing on bioethics mediation, it is necessary to test whether conceptual frameworks such as ours can also meaningfully be applied to detect more nuanced differences between specific models of CESS. Finally, the analysis presented in this paper does not give an account about “good” or “appropriate” evaluation. For such statement, it would be necessary to analyse the models as well as corresponding evaluation criteria with reference to relevant normative as well as empirical criteria. The methodological frameworks for so-called empirical-ethical analysis may provide a means to do so. However, given the considerable variations of CESS as well as other ethics activities in health care practice (e.g. ethics teaching or research ethics committees) and the complexity of these interventions, we believe that our work can serve as a starting point for future research in this respect.

## Conclusions

Even considering the limitations above, we conclude that using methodological approaches and tools of health service research provide new perspectives on the topic of evaluating outcomes of CESS. The development of a conceptual framework elucidates structural and procedural elements of a specific CESS, which are relevant for making decisions about outcome criteria. Moreover, beyond the field of outcome evaluation research, conceptual frameworks can facilitate shared understanding of the relevant elements and processes of a particular CESS and a deliberation about what it means to offer “good” CESS.

## Data Availability

Not applicable.

## Abbreviations

CESS: Clinical ethics support services
MCD: Moral case deliberation
MRC: Medical Research Council
